# Size development of tomatoes growing in trusses: linking time of fruit set to diameter

**DOI:** 10.1002/jsfa.10447

**Published:** 2020-05-15

**Authors:** L M M Tijskens, S van Mourik, J A Dieleman, R E Schouten

**Affiliations:** ^1^ Horticulture and Product Physiology Wageningen University and Research Wageningen The Netherlands; ^2^ Farm Technology Wageningen University and Research Wageningen The Netherlands; ^3^ Greenhouse Horticulture Wageningen University and Research Wageningen The Netherlands

**Keywords:** tomato, diameter, size, time of fruit set, truss

## Abstract

**BACKGROUND:**

Size of fruit is an important issue in determining yield at harvest. Even under controlled conditions, variation between fruit and trusses can be considerable. As an easy to measure indication of size, the diameter of tomatoes growing in trusses was assessed in three experiments with different number of tomatoes per truss, as well as cultivars, and also by varying the level of ions in the recirculated drain water.

**RESULTS:**

By applying the von Bertalanffy growth model, more than 99% of the variation present could be explained by the time of fruit set for all tomatoes growing anywhere in the trusses. A linear relationship between time of fruit set and the biological shift factor, an indication of developmental age, was observed. Integrating this linear relationship in the analysis of the diameter data removed one stochastic variable (biological shift factor), effectively halving the number of parameters to be estimated.

**CONCLUSION:**

The results of the present study indicate that the major part of the variation present in the diameter of tomatoes growing in trusses is the result of variation in the time of fruit set of individual fruits. The position within the greenhouse (i.e. local differences in assimilates supply) exerted only a minor effect on diameter development. Accordingly, the time of fruit set largely determines fruit size. Likely, growing conditions before fruit set are crucial for final fruit size. The time of fruit set of each tomato in the truss and the local growing conditions within the greenhouse that affect assimilate supply need to be assessed accurately for a reliable size prediction.

## INTRODUCTION

Fruit size is an important issue in horticultural production as it determines, together with crop load, fruit yield. Precise monitoring of the timing and quantification of yield is becoming increasingly feasible in greenhouse management as a result of the emergence of machine vision.^1^, ^2^, ^3^ Fruit size depends on many factors, including cultivar, foliar fertilisation,^4^, ^5^ irrigation,^6^ crop load^5^, ^7^ and weather conditions.^8^ Temperature has a major effect during the cell division period and probably also during formation in the meristem, but far less so during cell expansion period.^9^, ^10^, ^11^ The importance of temperature in this period has been related to the assimilate production. For the purpose of understanding and predicting size and yield, many fruit growth models have been developed. Some studies have included the generation of cells,^12^, ^13^ the time of flowering,^14^, ^15^ transport of assimilates from leaves to fruits,^16^, ^17^, ^18^, ^19^ cell elongation^20^ and cell expansion.^20^, ^21^ However, these models are not useful to growers with respect to optimising harvest timing and expected yield as a result of their complexity and over parametrisation. West *et al*.^22^ modelled mass increase based on fundamental principles of systems biology on the allocation of metabolic energy between maintenance and the production. For graphical representation of the generic growth curve, West *et al*.^22^ actually converted mass into diameter. Tran *et al*.^23^ measured the diameter of tomatoes, but they converted this into mass and modelled its behaviour using the empirical Gompertz curve. Tijskens *et al*.^11^ applied a simple modelling approach, based on a plausible kinetic mechanism, adapting and reformulating the von Bertalanffy growth model^24,25.^ The adaptation focussed on including the variation between individual fruits to describe the diameter growth of tomatoes, apples and pepper fruits. Size was expressed as fruit diameter, which is considerably easier to measure than mass or volume. Size, both measured and simulated, can be converted into volume assuming the appropriate spherical (tomato, apple) or cylindrical shape (pepper). For all fruit species studied, size increased along the same generic growth pattern with a single stochastic variable: the biological shift factor. This factor was used to describe the variation in size, provided the time of fruit set is measured. This adapted von Bertalanffy model described diameter growth accurately for individual fruits, with growth rate constants similar per species. In the present study, the adapted von Bertalanffy growth model is further developed towards describing diameter growth, not only for individual tomatoes, but also for tomatoes growing in trusses. This truss diameter model was used to explore how growth conditions and greenhouse management affect diameter of tomatoes growing in trusses. Diameter data were recorded of tomatoes, as cultivated in greenhouse compartments, that varied in truss thinning, as well as salinity levels in the recirculated drain water, and also comprised two experimental cultivars. The size of tomatoes in a truss can be increased by thinning after fruit set,^24^ or by limiting the number of fruit cells at floral development.^25^ Both processes rely on the availability photo assimilates, each in a different period of development. Sodium accumulation in the drain water is a common problem in greenhouse tomato cultivation limiting crop growth^26^ and the size of tomatoes,^27^ although it can also induce higher tomato quality.^28^ We aim to show that, by further adapting the von Bertalaffy model, the dynamics of diameter increase of tomato in trusses can be described accurately regardless of growth conditions and management. Steps to predict diameter at harvest for tomato trusses are discussed. For reliable predictions, it is crucial to measure fruit set accurately. Furthermore, it is indicated that techniques to assess the local assimilate supply need to be developed to accurately assess the diameter of tomatoes at harvest.

## MATERIALS AND METHODS

### Thinning experiment

Tomato plants (*Solanum lycopersicum* L., cv Komeett) were sown in week 32, 2015, grafted on a Maxifort rootstock and topped to give two stems per plant. In week 41, the plants were transplanted to a 144‐m^2^ greenhouse compartment (greenhouse A) at Wageningen UR Greenhouse Horticulture in Bleiswijk, The Netherlands. Crop management was according to commercial practise. Natural light was supplemented with 186 μmol m^−2^ s^−1^ LED lighting (Philips Greenpower, 95% red, 5% blue; Signify, Eindhoven, The Netherlands). When the outside solar radiation exceeded 600 W m^−2^, the lamps were switched off. Plants selected for diameter measurements were grown either at the left or right row of the greenhouse compartment. The right row was facing south, obtaining more sunlight than the left one. Trusses were thinned at flowering time of individual trusses to have two, four, five or six tomatoes per truss and labelled individually. From February until the end of April 2016, the date of fruit set was recorded and diameter assessed for each individual fruit.

### Cultivar experiment

Tomato plants of two experimental cultivars were sown in week 34, 2015 and transplanted in week 40 to a 144‐m^2^ greenhouse compartment (greenhouse B) of Wageningen UR Greenhouse Horticulture in Bleiswijk. Tomatoes of each cultivar were grown in separate plots in the greenhouse (Table 1). Natural light was supplemented with 125 μmol m^−2^ s^−1^ HPS lamps (Signify) and two LED interlighting modules (106 μmol m^−2^ s^−1^, Philips Greenpower; Signify). The date of fruit set of tomatoes in selected trusses was recorded and diameter assessed regularly for each individual fruit.

**Table 1:** Overview of the experimental set‐up

Green house	Cultivar	Row or treatment	Salinity (mmol L^−1^)				Fruit per truss	Number of plants	Number of fruit	Number of trusses	*N*_obs_
			Na	K	Ca	Mg					
A	Komeett	L					2	12	46	23	739
A	Komeett	L					4	17	124	31	1884
A	Komeett	L					5	11	100	20	1576
A	Komeett	L					6	4	30	5	564
A	Komeett	R					2	11	44	22	697
A	Komeett	R					4	13	104	26	1585
A	Komeett	R					5	9	85	17	1321
A	Komeett	R					6	7	54	9	993
B	cv1	L					6	17	176	30	2736
B	cv2	R					6	17	185	31	2849
C	Livento	A	5	8.0	9.5	2.8	6	9	54	9	1967
C	Livento	B	10	6.8	8.0	2.4	6	9	54	9	1915
C	Livento	C	15	5.5	6.6	1.9	6	9	54	9	1935
C	Livento	D	20	4.3	5.1	1.5	6	9	54	9	1820

L&R refer to trusses harvested on the left (L) or right (R) side of the greenhouse. *N*_obs_, number of observations.

### Salinity experiment

Tomato plants cv Livento were sown in early 2018, grafted on a Maxifort rootstock and transplanted to a greenhouse compartment of 144 m^2^ at Wageningen UR Greenhouse Horticulture (greenhouse C) in Bleiswijk without supplemental lighting. Four treatments were applied varying the level of ions (Na, K, Mg and Ca) in the recirculated drain water but retaining a constant electrical conductivity of 3.8 mS cm^−1^ (Table 1). Each treatment consisted of nine plants in one gutter, randomly assigned to a location within the greenhouse compartment. Between 16 April and 6 June 2018, the date of fruit set was recorded for each individual fruit and trusses were labelled.

### Fruit set, fruit number and diameter measurements

Fruit set was defined as the time, individual for each flower, at which all petals dropped off when tapping the flower gently by hand. Fruit numbering in a truss started with the tomato with the earliest fruit set as number one and continued in sequence. Starting from day five after fruit set, every 2–3 days, the diameter of all fruit was measured at the equator of the fruit at the highest diameter with a digital calliper (S_Cal EVO BT; Sylvac S.A., Crissier, Switzerland). Every hour, the calliper was washed with water to remove deposited plant parts. Diameter measurements continued until size increase levelled off, as assessed by visual observation. Most, but not all tomatoes, were red coloured at that time. Table 1 provides an overview of the experimental set‐up with number of plants, number of fruit and number of observations. Time of measurement of each fruit was expressed counting from the time of fruit set of the first tomato in that truss.

### The adapted von Bertalanffy model

**Figure 1:**
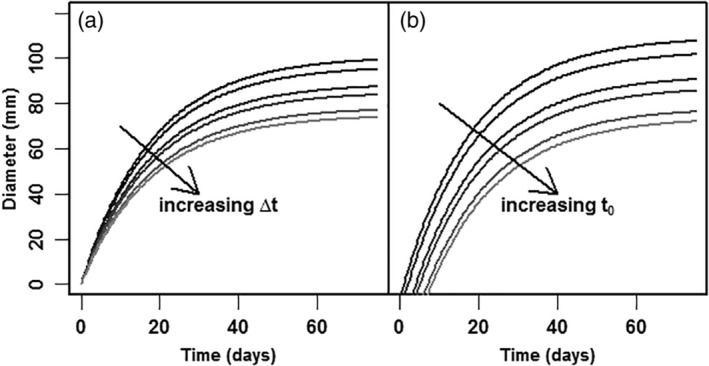
An example for the effect of variation in biological shift factor (a: *Δt* ) and fruit set (b: *t*_0_ ). Parameter values are taken from the results of the analysis of cv Komeett greenhouse A row R, all trusses.

The model, as applied throughout the present study, is the adapted von Bertalanffy model, as developed and described in Tijskens *et al*.^11^ In short, von Bertalanffy^29^, ^30^ proposed a model showing an exponential increase towards a maximum size. This model was adapted and reformulated to include the variation in growth of individual fruits, as shown in Eqn (1):

(1) $$D\left(t\right)=D_{\mathrm{ref}}\cdot \left(e^{-k\cdot \left(t_{0}+\mathrm{\Delta t}\right)}-e^{-k\cdot \left(t+\mathrm{\Delta t}\right)}\right)$$

with *t* the time after fruit set of the first tomato in the truss, *D(t)* the diameter (in mm) at time *t* (in day), *t*_0_ (in day) the time of fruit set (i.e. the time for each individual fruit in the truss at which diameter is zero), and *k* (day^−1^) is the growth rate constant. *Δt* (in days) is the biological shift factor, a stochastic variable, different for each individual tomato and indicative of the stage of maturity. The biological shift factor actually indicates the time of development for each individual fruit along the generic growth curve, *D*_ref_ is a reference diameter, set at 40 mm for all experiments, which is roughly mid‐range. The deduction of the model and the definition of Δ*t* is presented in the Supporting information Mathematical deductions. In Fig. 1, the effect of variation in biological shift factor (*Δt*) and time of fruit set (*t*_0_) is shown for simulated data. The biological shift factor (*Δt*) affects the maximum diameter, while the time of fruit set (*t*_0_) in addition to modify the maximum diameter, predominantly causes a shift over the time axis. At infinite time, the equation reduces to Eqn (2):

(2) $$D_{\infty }=D_{\mathrm{ref}}\cdot e^{-k\cdot \left(t_{0}+\Delta t\right)}$$

where *D*_*∞*_ is the maximum diameter at infinite time, an indication for the size at harvest. For graphic representations, the size development can be expressed in a standardised form (*D*_stan_) to observe the generic behaviour by dividing both sides of equation Eqn (1) by the asymptote value *D*_*∞*_ in Eqn (2):

(3) $$\underset{D_{\text{stan}}}{\underset{︸}{\frac{D\left(t\right)}{D_{\infty }}}}=1-e^{-k\cdot \left(t-t_{0}\right)}$$

Equation (3) effectively normalises all diameter values between 0 and 1. All variation as a result of biological shift factor (*Δt*) is now contained on the left side, whereas all variation as a result of the time of fruit set (*t*_0_) is contained on the rightside. Eqn (3) represents the generic development pattern of growing fruits.^31^ The term *t – t*_0_ represents the development or biological time subsequent to fruit set for each individual fruit in a truss.

During data analysis, the estimated biological shift factor (*Δt*) is found to be linearly related to the estimated time of fruit set (*t*_0_) according to Eqn (4):

(4) $$\mathrm{\Delta t}=\beta_{0}+\beta_{1}\cdot t_{0}$$

where β_1_(−) is the slope and β_0_(day) is the intercept. The slope (*β*_1_) could be taken in common for all trusses, whereas the intercept *β*_0_ differed per individual truss.

### Statistical analysis

Model development and mathematical deductions were conducted in Maple 2016 (MapleSoft, Waterloo Maple Inc, Waterloo, Canada), a computer program capable of handling and solving algebraic and differential equations. Data on diameter were analysed without transformation based on Eqn (1) using indexed non‐linear regression analysis^32^ and applying the procedure ‘nls’ of the statistical package R.^33^ Indexed means that some parameters (*k, β*_1_) were estimated in common for all fruit (fixed effects), whereas other parameters were estimated as stochastic parameters (random effects) for each fruit (*Δt* and *t*_0_) or for each truss (*β*_0_). Testing the normality of the obtained distributions was conducted using the Shapiro–Wilk test in R.

## RESULTS

### Analysis of the diameter data with the adapted von Bertalanffy model

**Figure 2:**
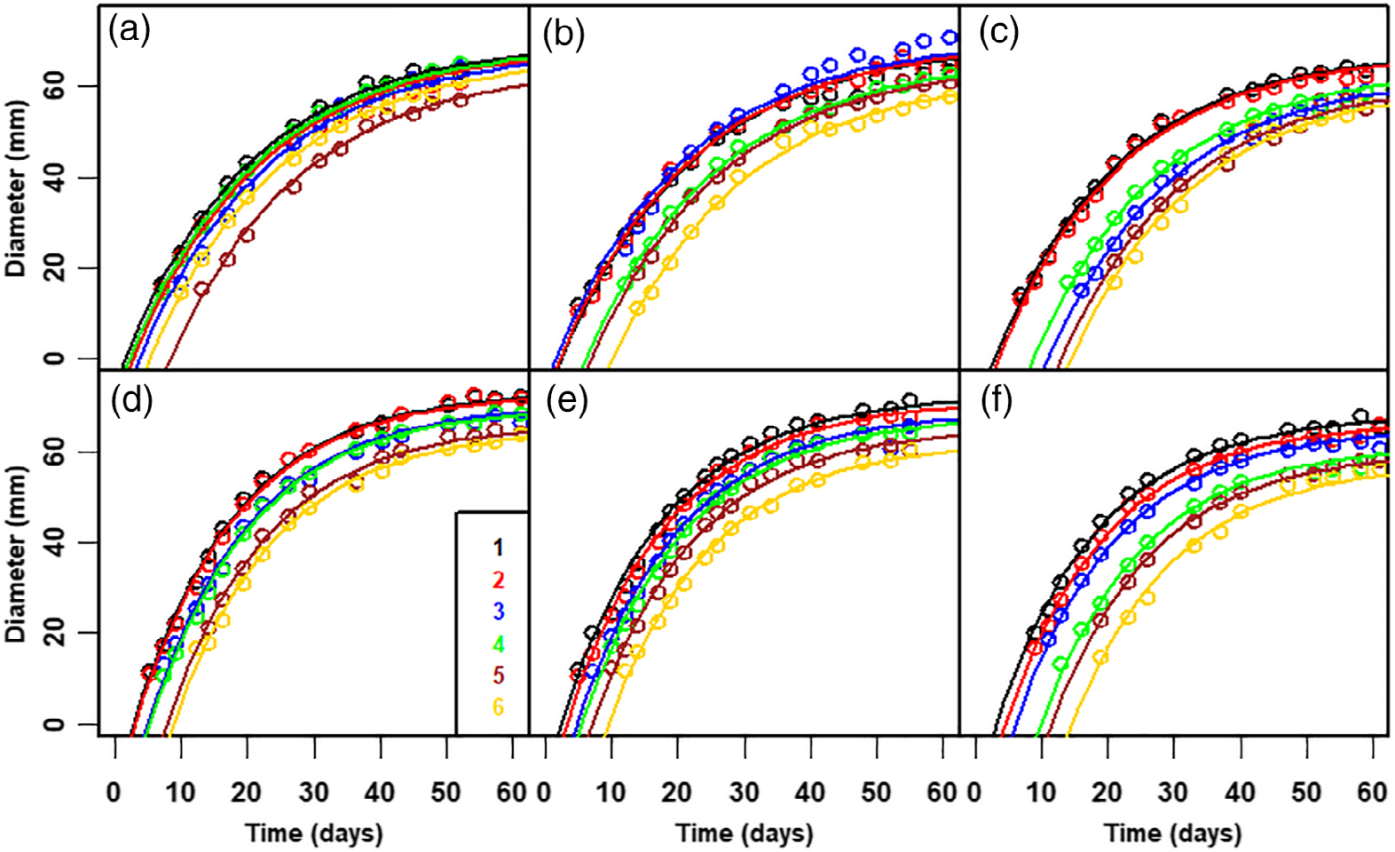
Raw data and model simulation of the diameter dynamics of all tomatoes in a truss *versus* time *t* (subsequent to fruit set of first fruit in the truss). (a–c) cv Komeett (greenhouse A). (d–f) cv 1 (greenhouse B). Each graph covers all tomatoes in a single truss. Trusses are selected from the measured data to cover the wide range in time of fruit set. Lines are based on the results of nonlinear regression analysis (Tables 2 and 3). Symbol colours refer to successive tomatoes in the truss as indicated.

**Figure 3:**
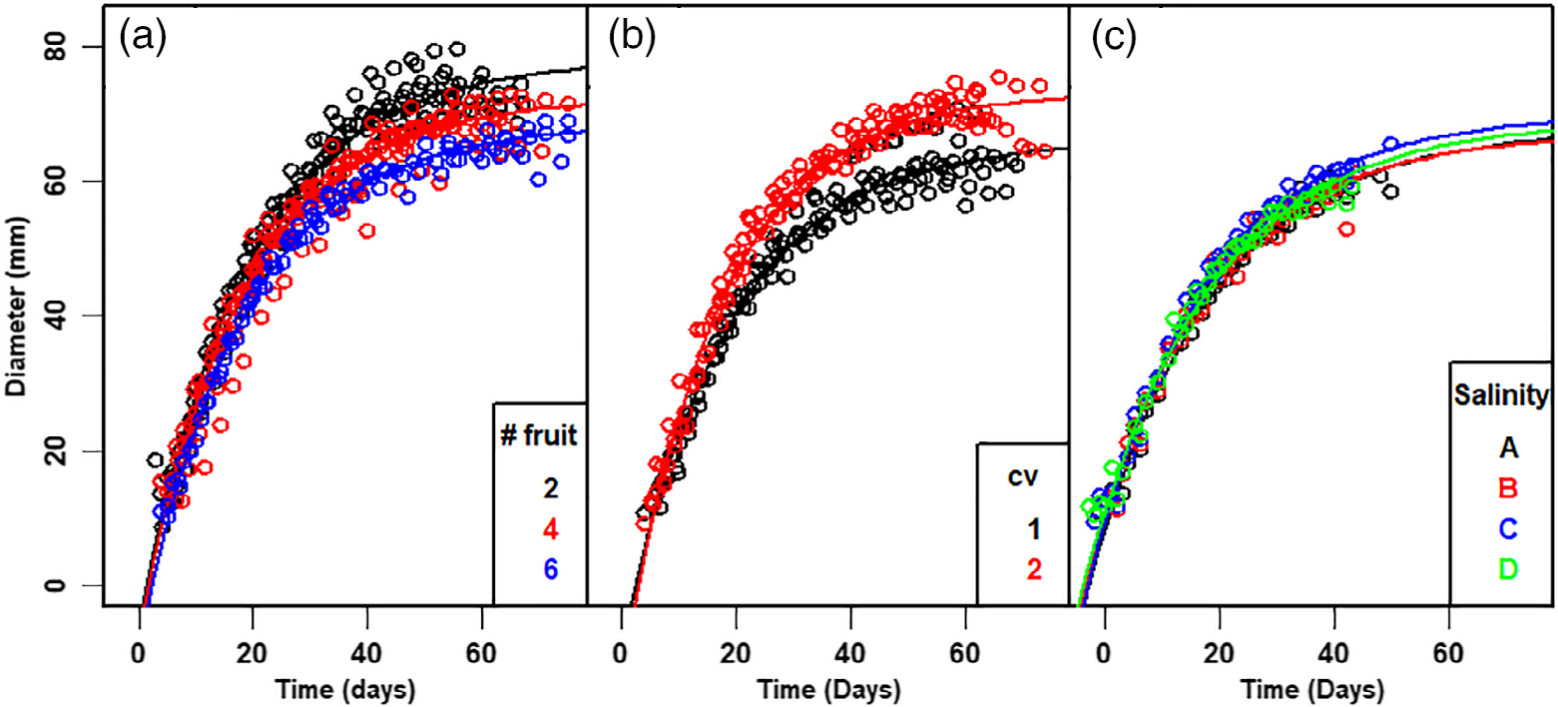
Raw data (mean per time point) and model simulation of the diameter dynamics of first fruit in a truss *versus* time *t* (subsequent to fruit set of first fruit in the truss). (a) cv Komeett greenhouse A, indicating the effect of thinning on size and behaviour, (b) experimental cultivars 1 & 2 greenhouse B and (c) cv. Livento greenhouse C, indicating the effect of the four levels of salinity treatment. Lines are based on the results of nonlinear regression analysis. Symbols refer to successive treatments as indicated.

The raw data showed large variation in fruit diameter, between fruit, even within the same truss. Nevertheless, fruit diameter during growth showed the same pattern for all tomatoes, irrespective of treatment (Fig. 2). Time of fruit set for all tomatoes in a truss (the intercepts on the time axis in Fig. 2) varied between five (Fig. 2d) and 15 days (Fig. 2f). The maximum diameter achieved is larger for trusses with two tomatoes than for trusses with four or six fruit (Fig. 3a). For the salinity experiment, only small differences were observed between treatments (Fig. 3c).

**Figure 4:**
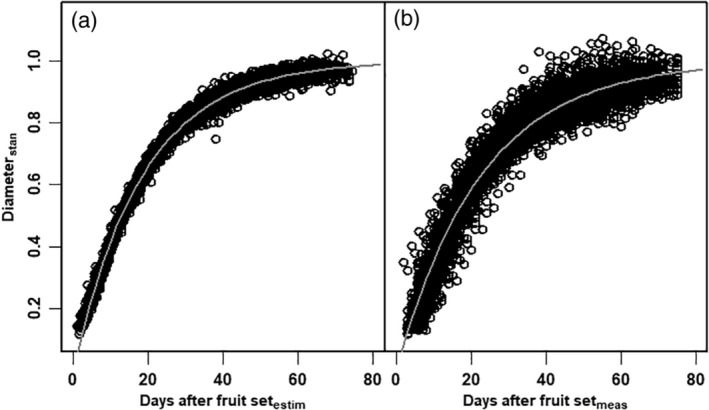
Standardised diameter (Eqn 3) of all tomatoes of cv Komeett (greenhouse A) *versus* time subsequent to fruit set of each fruit (*t* − *t*_0_), indicating the generic applicability of the adapted von Bertalanffy growth model. (a) Standardised diameter with *t*_0_ the estimated time after fruit set, (b) Standardised diameter with *t*_0_ the measured time after fruit set.

**Figure 5:**
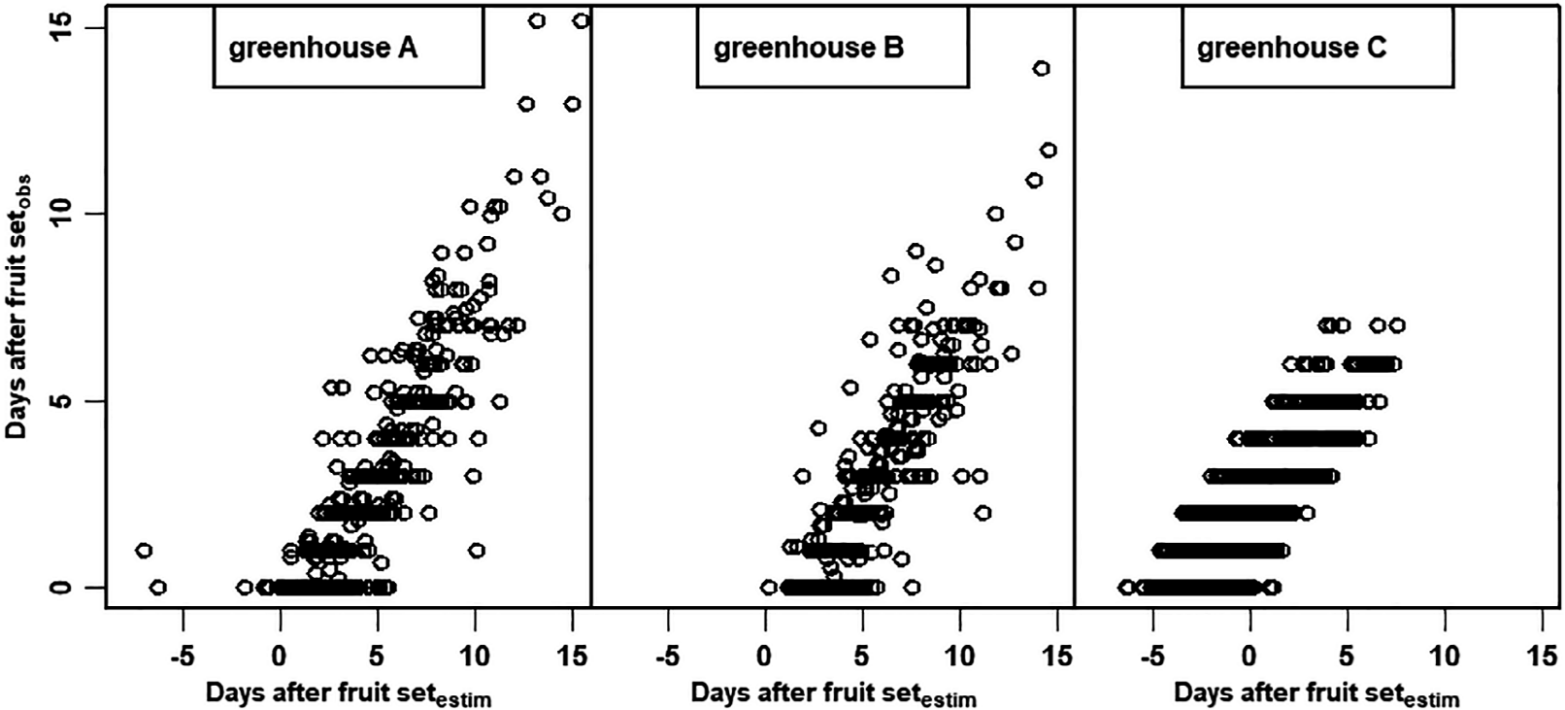
The observed time of fruit set (*t*_0_) *versus* the estimated time after fruit set (*t*_0_) for all fruit in greenhouse A, B and C.

Diameter data were analysed using indexed nonlinear regression based on Eqn (1), estimating the biological shift factor (*Δt*) and the time of fruit set (*t*_0_) for each individual tomato, applying a common rate constant of size increase (*k*) for all tomatoes in a treatment (Table 2). This analysis provides an estimated value for each fruit of the time of fruit set (*t*_0_) and the biological shift factor (*Δt*). The percentage variance accounted for by the model, and adjusted for the number of independent variables (*R* ^2^_adj_) was higher than 99% for all treatments. Simulated diameter behaviour for a number of selected tomato trusses, applying the model parameters of Table 2 are shown in Fig. 2. Experimental and simulated standardised diameter (Eqn 3) values of all left row Komeett tomatoes are shown in Fig. 4(a), which indicates the generic applicability of the adapted von Bertalanffy growth model because it contains all tomatoes of one cultivar, each with its own time of fruit set and biological shift factor. The rate constants per treatment are very similar, irrespective of the number of fruits in a truss or salinity treatment. The observed *versus* the estimated time of fruit set per tomato is shown in Fig. 5.

**Table 2:** Results of the indexed nonlinear regression analysis, estimating the biological shift factor (Δ*t*) and the time of fruit set (*t*_0_) for each individual fruit applying Eqn (1) with indicated standard deviation (SD) and standard error of estimation (SEE)

Experimental Set‐up/administration						Parameters									
Green house	Row/Treatment	cultivar	Fruit/truss	Number of fruit	*N*_obs_	*k* (d^−1^)		*Δt* (d)			*t*_0_ (d)			*D*_*∞*_ (mm)	
						Value	SEE %	Average	SD	SEE %	Average	SD	SEE %	Average	SE
A	L	Komeett	2	46	739	0.051	0.7	−15.2	1.5	1.9	2.23	1.04	9.0	77.5	4.5
A	L	Komeett	4	124	1884	0.052	0.4	−14.4	2.2	1.9	3.81	2.81	5.2	69.7	4.0
A	L	Komeett	5	100	1576	0.051	0.5	−14.9	2.6	2.1	4.65	3.23	5.0	67.5	5.5
A	L	Komeett	6	30	564	0.053	0.8	−15.1	2.7	1.9	5.74	3.27	3.8	65.7	3.8
A	L	Komeett	All	300	4763	0.052	0.3	−14.8	2.3	2.0	4.03	2.96	5.2	69.7	5.8
A	R	Komeett	2	44	697	0.055	0.6	−13.3	1.8	1.8	1.48	1.89	11.5	76.9	4.5
A	R	Komeett	4	104	1585	0.057	0.5	−14.0	2.0	2.0	3.54	2.31	5.8	72.5	3.5
A	R	Komeett	5	85	1321	0.055	0.6	−14.0	2.2	2.3	4.21	2.44	5.6	68.7	4.2
A	R	Komeett	6	54	993	0.056	0.6	−14.6	3.1	2.1	5.99	3.65	3.8	65.1	3.3
A	R	Komeett	All	287	4596	0.056	0.3	−14.0	2.3	2.0	3.88	2.90	5.4	70.5	5.5
B	L	cv1	6	176	2736	0.055	0.5	−13.7	2.5	3.2	5.01	2.50	5.5	64.6	3.7
B	R	cv2	6	185	2849	0.058	0.5	−15.6	2.6	2.6	6.10	3.39	4.8	70.1	4.8
C	A	Livento	6	54	1967	0.050	0.7	−13.2	2.4	3.4	3.40	2.70	8.2	65.3	3.3
C	B	Livento	6	54	1915	0.052	0.7	−12.7	2.3	3.6	3.50	2.54	8.1	64.6	3.1
C	C	Livento	6	54	1935	0.050	0.7	−13.8	2.6	3.5	3.01	2.67	9.9	68.5	3.2
C	D	Livento	6	54	1820	0.053	0.8	−12.2	2.4	5.2	3.42	2.65	10.1	64.3	3.6

The reference diameter (*D*_ref_) (Eqn 1) is set to 40 mm for all treatments. The diameter at infinite time (*D*_*∞*_) is calculated (Eqn 2) based on the estimated parameter values. *N*_obs_, number of observations.

### Time of fruit set and biological shift factor are linearly related

**Figure 6:**
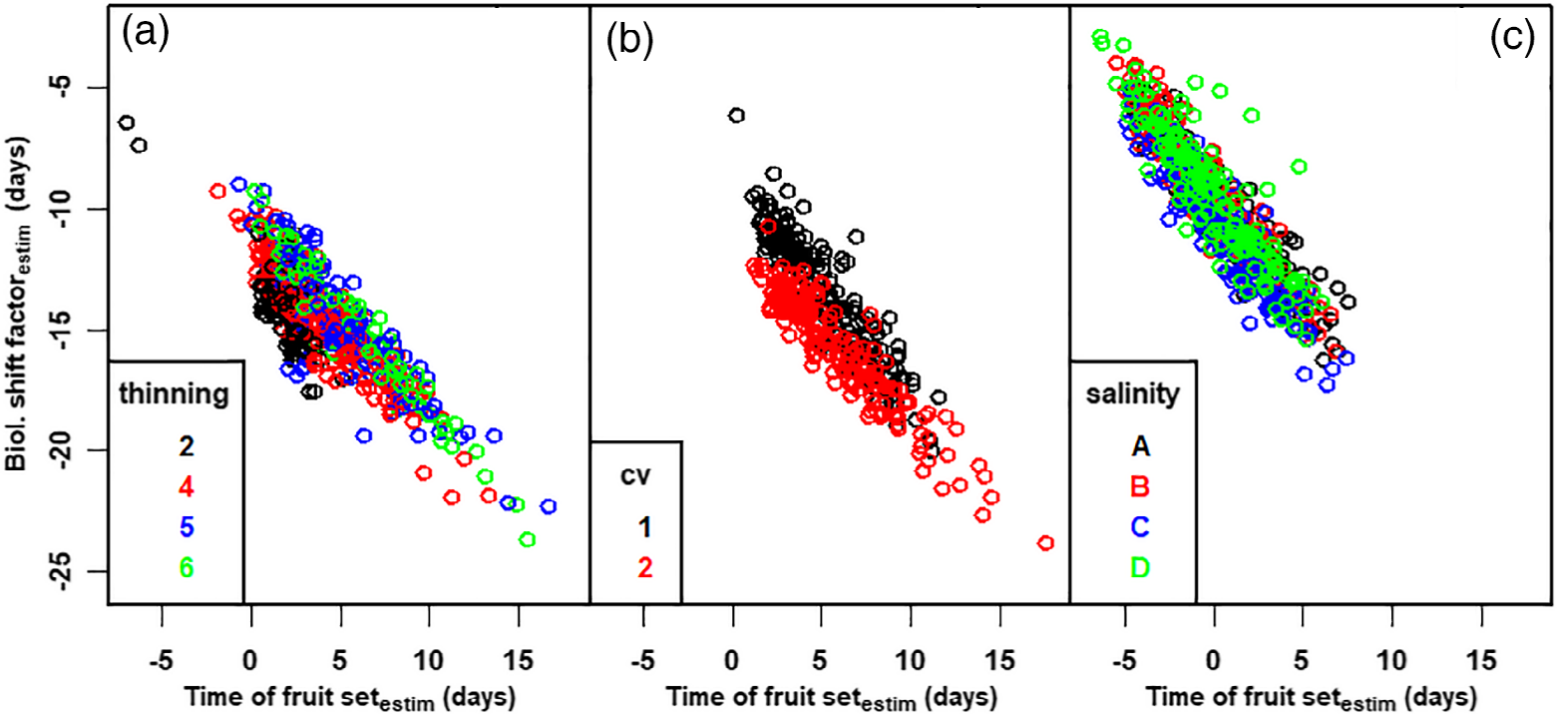
The estimated biological shift factor (*Δt*) *versus* the estimated time of fruit set (*t*_0_). (a) all fruit in thinning experiment (cv Komeett greenhouse A) for the different thinning levels, (b) all fruit in the cultivar experiment (greenhouse B) and (c) all fruit in the salinity experiment (cv Livento greenhouse C).

**Figure 7:**
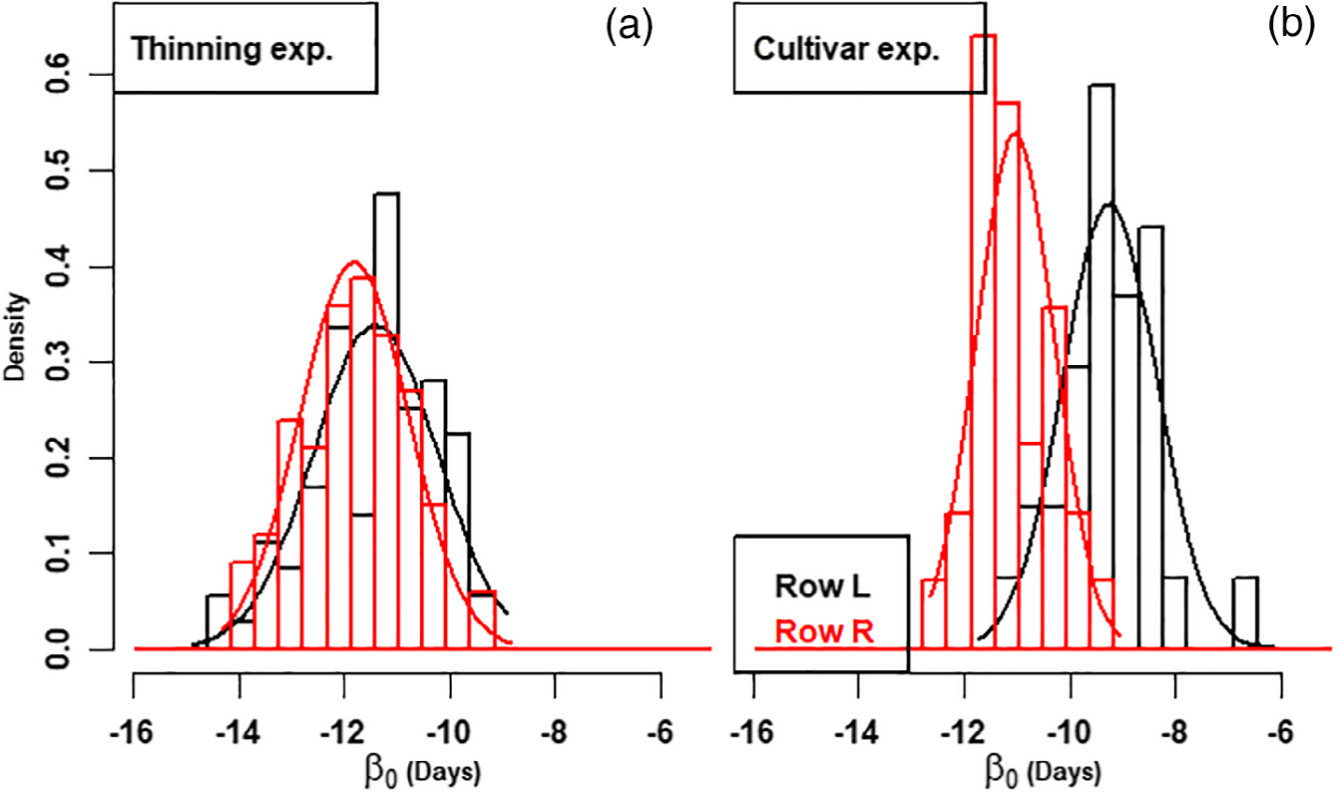
Distributions of *β*_0_ per row in the greenhouse. (a) All tomato trusses of the thinning experiment in greenhouse A and (b) All tomato trusses of the cultivar experiment greenhouse C. Density graphs are similar to histograms. To avoid the effect of differences in number of observations between the samples, the *y*‐axes are expressed as the occurring density.

Within a truss, the fruit with the latest fruit set has the smallest maximum diameter (Fig. 2). This indicates that the stochastic variables (*Δt* and *t*_0_) are expressions of the same source of variation. The estimated biological shift factor (*Δt*) *versus* the estimated time of fruit set (*t*_0_) per greenhouse reveals a strong linear relationship (Fig. 6). The width of cloud of points in Fig. 6 indicates additional variation. Within treatments, the linear relationship per truss has approximately the same slope but with an intercept varying per truss (Eqn 4). An example is shown in the Supporting information (Fig. S1). All other combinations show a highly similar pattern. This means that all diameter data can be analysed as before, based on Eqn (1), but now incorporating a linear relationship between the biological shift factor (*Δt*) *versus* the estimated time of fruit set (*t*_0_), as shown in Eqn (4). The results are shown in Table 3. The rate constant of size increase (*k*) and the slope (*β*_1_) were estimated in common for all tomatoes in a greenhouse, whereas the intercept *β*_0_ was estimated per truss. The time of fruit set (*t*_0_) was used as estimated per individual tomato in the initial analysis (Table 2). The value for *R* ^2^_adj_ was only slightly lower than before: about 98.7% for all treatments (Table 3). By correcting the biological shift factor (*Δt*) (i.e. by subtracting the variable *β*_*0*_ per truss and adding the mean value), the relationship between *t*_0_ and *Δt* improved considerably (see Supporting information, Fig. S2). Because the stochastic variables (*Δt* and *t*_0_) are estimated using all time points simultaneously, the variables and especially the distributions are independent of the time of development and therefore most probably normally distributed. The normality of the *β*_*0*_ distributions (Fig. 7) could indeed not be rejected according to the Shapiro–Wilk test (*P* > 0.05).

**Table 3:** Results of the analysis incorporating the linear relationship between time of fruit set (*t*_*0*_) and biological shift factor (*Δt*) (Eqn 4)

					Parameters												
Experimental setup/Administration					*k* (d^−1^)		*β*_0_ (d)			*β*_1_ (−)		*D*_*∞*_ (mm)			Volume (cm^3^)		
Green house	Row/Salinity	R^2^_adj_	N_obs_	Number trusses	Value	SEE %	Value	SD	SEE %	Value	SEE %	Value	SD	SE %	Value	SD	SE %
A	L	0.987	4763	79	0.052	0.317	−12.09	1.23	0.96	−0.74	0.62	69.68	5.28	7.58	57.38	13.26	23.11
A	R	0.987	4596	74	0.056	0.314	−11.17	0.99	0.95	−0.80	0.58	70.45	4.97	7.05	59.16	12.55	21.22
B	L	0.989	2736	30	0.055	0.385	−9.21	0.90	1.18	−0.89	0.63	64.64	3.25	5.02	45.35	6.73	14.84
B	R	0.988	2849	31	0.058	0.376	−10.99	0.70	0.86	−0.76	0.57	70.06	4.45	6.35	58.00	10.37	17.88
C	All	0.987	7637	108	0.051	0.319	−9.68	0.85	1.22	−0.86	0.47	65.65	2.78	4.24	47.41	5.95	12.54

*t*_0_ was taken as estimated in previous analysis (Table 2). Rate constant (*k*) and slope (*β*_1_) were estimated in common for all fruit in the set, whereas *β*_0_ was estimated per individual truss. *N*_obs_, number of observations.

### Applying the measured time of fruit set

To increase the practical applicability of the presented model and the simplicity of the measuring system, the time of fruit set *t*_*0*_ was not estimated, but the visually observed values per tomato were used (Eqn 1). Applying these measured times of fruit set, the estimated model parameters (Table 3) were similar to those shown in Table 2. The SEEs of estimates are somewhat larger and the value for *R* ^2^_adj_ was slightly lower but well above 98% for all treatments.

## DISCUSSION

### Time of fruit set: observed *versus* estimated

A plot of the observed *versus* the estimated time of fruit set per tomato for the analysis (Table 2) shows a high variability (Fig. 5). The variation of the intercept in these graphs reflect the uncertainties in assessing and recording when the first fruit set occurred, whereas the cloud of points expresses the irregularities due to irregular observation frequency. The frequency of observation was rather low, every 2–3 days. The time of fruit set was recorded by the same observer in greenhouses A and B but by another observer in greenhouse C. Although both observers used the same definition of fruit set (the time that the flower petals fall off when tapping the flower gently), the first observer appears to overestimate and the second observer appears to underestimate the time of fruit set (see intercepts in Fig. 5). This variability in determining time of fruit set resulted in a much higher uncertainty in the simulated diameter values compared to applying estimated times of fruit set (Fig. 4). Dorey *et al*.^14^ also reported on the importance of an accurate determination of the time of flowering (fruit set). Determining fruit set accurately is therefore of importance with regard to practical implementation of the adapted von Bertalanffy model. Recent developments in machine vision focus on identifying shape, size and maturity of the tomatoes ready to be harvested.^1^, ^2^, ^3^ This means that automated size measurements during growth is almost becoming reality.^34^ If diameter growth can be measured with a high frequency (e.g. every hour), the time of fruit set can be deduced accurately. Machine vision might also be used to assess the time of fruit set by monitoring tomato flower petals.

### Thinning affects the maximal diameter of tomatoes

Fruit in a truss are set in sequence, with sometimes large differences in time of fruit set within a truss (Fig. 2). Within a truss, flowers and peduncles are generated in the meristem.^12^, ^21^ It is likely that, for this process, the level of assimilates at the time of fruit set is most important, and conceivably even earlier,^35^ although far less during the period of actual growth.^9^, ^10^, ^12^, ^25^, ^36^ The term sink strength was defined as the competitive ability of an organ to attract assimilates.^16^ Lowering the sink strength of a truss by fruit thinning causes more assimilates to be available for the remaining fruits and vegetative parts. Indeed, with thinning, the final diameter increased. The difference in final diameter between trusses with two and six fruits was approximately 12 mm (Fig. 3 and Table 2), resulting in an approximately 50% larger volume, assuming tomato volume can be described as a sphere. In other words, a small increase in final diameter (*D*_∞_) with increased thinning, has a large effect on volume. This means that any inaccuracy in measuring diameter due to measuring error or observer interpretation of fruit set (Fig. 5) is amplified greatly, resulting in much higher inaccuracies when deducing the volume of fruits. This stresses the importance of an accurate measurement not only for achieving accurate diameter predictions, but also especially when converting to volume.

The results of the salinity experiments (greenhouse C) were only very slightly different for the four salinity treatments. The standard deviation (SD) of time of fruit set, the biological shift factor and the diameter at infinite time (*D*_∞_) were similar (Table 2). At this level of electro conductivity, changes in salinity do apparently not affect growth of tomatoes.

### Size increase modulated by assimilate supply?

During the first analysis, a linear relationship was found between the estimated time of fruit set and the estimated biological shift factor (Fig. 6), showing that younger fruit (late fruit set) are less mature (lower biological age) and *vice versa*. Likely, both stochastic variables (*t*_0_ and *Δt*) are expressions of the same underlying variation. For all tomatoes, the rate constant of diameter increase (*k*) and the slope (*β*_1_) were found to be very similar for all treatments, cultivars and greenhouses. This allowed one stochastic parameter, *Δt*, to be replaced by a common slope (*β*_1_) and one stochastic parameter estimated per truss, the intercept (*β*_0_). Although one parameter, *Δt,* is replaced by two new parameters, the model is actually simplified with more degrees of freedom as *β*_*0*_ is estimated per truss instead of *Δt* per fruit. This reduced the number of parameters to be estimated considerably because there are more fruit than trusses, thus avoiding over‐parametrisation.

This linear relationship between the time of fruit set and the biological shift factor is not at all unexpected. Both variables express development in the time domain. The slope (*β*_1_), common to all fruit, is the conversion factor to express time of fruit set in time of diameter development. The intercept (*β*_0_), specific for each truss, is more difficult to interpret. The *β*_0_ distributions showed a clear difference per row and per greenhouse (Fig. 7). A higher value for *β*_0_ means a higher value for the biological shift factor, which indicates more mature fruit. The average values of *β*_0_ distributions varied with the position in the greenhouse (Table 3). In the thinning experiment, the right row was closer to the greenhouse exterior, receiving more sun exposure than the left row. In the cultivar experiment, the left row was closer to the greenhouse exterior. The side in each greenhouse with more sun exposure showed higher *β*_0_ values, indicative of more mature fruit. Because greenhouse temperature is controlled between strict boundaries, it appears that predominantly light conditions affect *β*_0_, possibly by affecting the photo assimilate supply locally available for diameter growth. Assimilates transported to organs have been shown to be predominantly generated at nearby sources (i.e. the leaves).^36^ Moreover, any transport by diffusion (either active or passive) is fast on short distance but slow on long distance.^37^ That makes it likely that assimilates used in size increase are just short distance transported. *β*_0_ might therefore be considered as related to the local assimilate supply (i.e., the actual amount of assimilates available for generation and development of trusses and fruit). The interpretation of *β*_0_ could be clarified by measuring diameter dynamics in experiments set up to affect local source‐sink ratios,^38^ by local leaf pruning and fruit thinning. Provided that the time of fruit set can be measured or deduced accurately, *β*_0_ is the sole stochastic variable in the model. To arrive at reliable diameter predictions of truss tomatoes, it is of prime importance to determine the relationship between *β*_0_ and the local assimilate supply, and to assess its magnitude. Indeed, the stochastic variable *β*_0_ most probably contains effects of local growing conditions. Because its effect is rather small (see the difference in the Supporting information between Fig. S1 and S2), it might be assumed that the major source of differences in size within and between trusses is generated during the cell division period, conceivably even earlier.

## CONCLUSIONS

Diameter dynamics of truss tomatoes comply with the adapted von Bertalanffy growth model, irrespective of the thinning, salinity treatments or cultivar. By estimating the time of fruit set and the biological shift factor per individual fruit, the percentage explained was higher than 99% for all series and treatments, with the same rate constant of diameter growth per cultivar. A considerable discrepancy exists between the estimated and the observed time of fruit set. The frequency of determining and recording the time of fruit set was likely too low. Moreover, a clear effect of observer was noticed. Applying the measured time of fruit set instead of the estimated time of fruit set leads to a slightly lower performance of the model.

A linear relationship was observed between the estimated time of fruit set and the biological shift factor. Within a treatment, the linear relationship was observed to have the same slope but an intercept varying per truss. Analysis of the diameter data incorporating this linear relationship revealed some variation in the intercept (*β*_0_) values. Variation in *β*_0_ might be interpreted as the effect of variation in locally available source strength within the greenhouse. The consequence is that more attention should be devoted to accurately measure the time of fruit set (e.g. by machine vision) and to determine the variation in *β*_0_ as related to the l*ocal assimilate supply* within the greenhouse to be able to predict the diameter, and subsequently volume, of tomato trusses at harvest.

## Supporting information


**Appendix** S1. Supporting information.
