# Supplementary material for: Size development of tomatoes growing in trusses: linking time of fruit set to diameter
Source: J Sci Food Agric. 2020 May 15;100(10):4020–8. doi: 10.1002/jsfa.10447 (PMC7384071; doi:10.1002/jsfa.10447)
Supplement: Supplementary file 1 — Appendix S1. Supporting information. [file JSFA-100-4020-s001.doc]

# Supplementary Material

# Mathematical deductions

The proposed mechanism is a simple first order production as depicted in Eq. SM1:

Eq. SM1

with S a substrate and D the product of the reaction (here the diameter), and k the reaction rate constant. Based on the rules of chemical kinetics the differential equations can be deduced:

$\begin{matrix} \frac{\partial S\left(t\right)}{\partial t}=-k\cdot S\left(t\right) \\ \frac{\partial D\left(t\right)}{\partial t}=k\cdot S\left(t\right) \end{matrix}$ Eq. SM2

With the initial conditions S(0)=S_0_ and D(0)=D_0_, the standard solution for this set of differential equations is, i.e., without taking biological variation into account:

$\begin{matrix} S\left(t\right)=S_{0}\cdot e^{-k\cdot t} \\ D\left(t\right)=D_{0}+S_{0}\cdot\left(1-e^{-k\cdot t}\right) \end{matrix}$ Eq. SM3

To convert the initial condition S_0_ into a shift in time (∆t), the integration constant can be formulated as S(-∆t)=S_ref_, with S_ref_ the reference value for ∆t. This constitutes at the same time the definition of the biological shift factor ∆t. Integration of the differential equations (Eq. SM2) then results in Eq. SM4:

$\begin{matrix} S\left(t\right)=S_{ref}\cdot e^{-k\cdot\left(\Delta t+t\right)} \\ D\left(t\right)=D_{0}+S_{ref}\cdot\left(e^{-k\cdot\Delta t}-e^{-k\cdot\left(\Delta t+t\right)}\right) \end{matrix}$ Eq. SM4

Since the time in these equations (and in the experimental setup) is rather arbitrarily chosen, the value of D_0_ will depend on that choice: when the experiment would have started earlier the value for D_0_ would be smaller. Assuming that the same mechanism is at work in the time before the start of the experiment, the time shift for the starting time of fruit growth (t_0_) can be deduced: at that point in time, the amount of product D will be zero. In terms of integration conditions that is D(t_0_)=0. This results in the analytical solution as shown in Eq. SM5. Since nothing has changed with respect to S(t), that result is the same as in Eq. SM4

$D\left(t\right)=S_{ref}\cdot\left(e^{-k\cdot\left(\Delta t+t_{0}\right)}-e^{-k\cdot\left(\Delta t+t\right)}\right)$ Eq. SM5

In the derived model, and its application in this paper, the substrate is considered nor measured, and all substrate S is ultimately converted into product D. The reference variable S_ref_ can therefore be replaced by D_ref_, resulting in the applied formulation:

$D\left(t\right)=D_{ref}\cdot\left(e^{-k\cdot\left(\Delta t+t_{0}\right)}-e^{-k\cdot\left(\Delta t+t\right)}\right)$ Eq. SM6

In all these equations t+Δt represent the age of each individual fruit, quite comparable to age of human beings. That is also not the same for each and every person.

# Additional figures


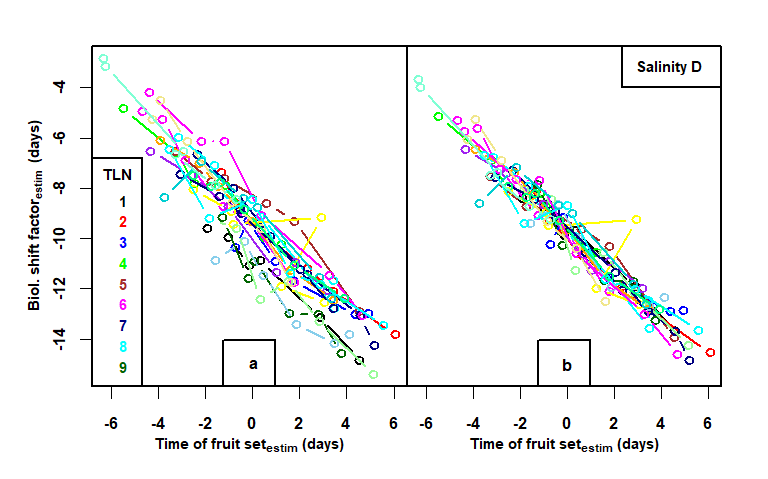


Figure S1 The linear relation between estimated biological shift factor (*Δt*) and estimated time of fruit set (*t_0_*) for salinity treatment D per truss location in the greenhouse (TLN). a: biological shift factor *Δt* as estimated, b: with intercepts corrected for *β_0_* (Eq. 4).


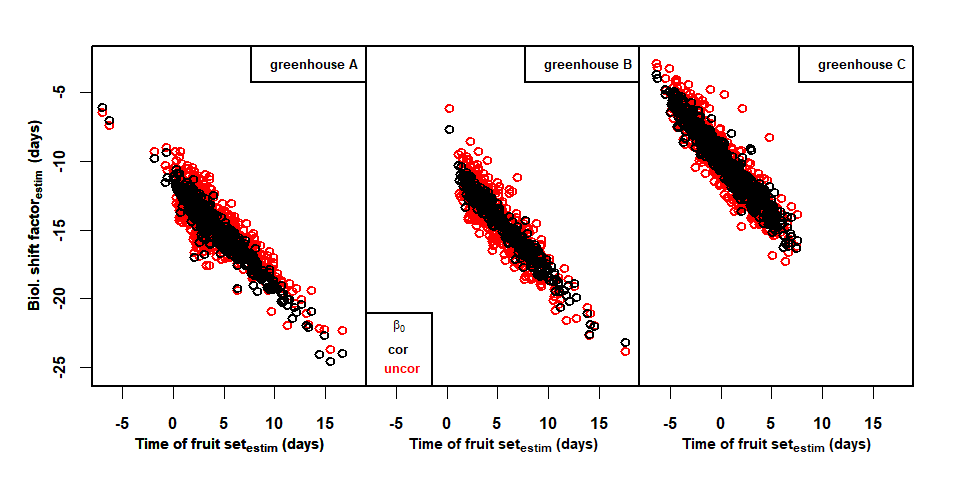


Figure S2 Estimated biological shift factor (*Δt*), corrected (black) and uncorrected (red) for the slope *β_0_* versus the estimated time of fruit set (*t_0_*) for all treatments in all three greenhouses.
